# Dichloridobis(methyl­amine-κ*N*)boron(III) chloride

**DOI:** 10.1107/S1600536808003589

**Published:** 2008-02-13

**Authors:** Markus Weinmann, Jürgen Nuss, Martin Jansen

**Affiliations:** a Max-Planck-Institut für Festkörperforschung, Heisenbergstrasse 1, 70569 Stuttgart, Germany

## Abstract

The title compound, C_2_H_10_BCl_2_N_2_^+^·Cl^−^ or [BCl_2_(H_3_CNH_2_)_2_]^+^·Cl^−^, is the first crystallographically characterized di(alkyl­amine)–BCl_2_^+^ salt. The B atom is tetra­hedrally coordinated by two Cl and two methyl­amine N atoms. In the crystal structure, the cations and anions inter­act *via* N—H⋯Cl hydrogen bonds (mean H⋯Cl = 2.40 Å), resulting in a layered structure.

## Related literature

For more details of the synthesis and background, see Weinmann, Nuss *et al.* (2007 ▶); Weinmann, Kroschel *et al.* (2007 ▶). For related structures, see: Nöth & Lukas (1962 ▶); Mikhailov *et al.* (1964 ▶); Nöth *et al.* (1966 ▶); Ryschkewitz & Myers (1975 ▶).
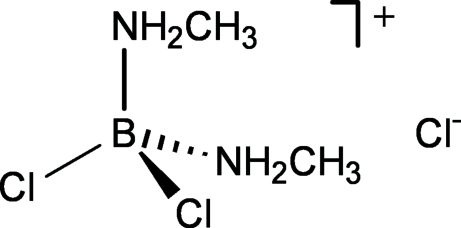


## Experimental

### 

#### Crystal data

C_2_H_10_BCl_2_N_2_^+^·Cl^−^
*M* *_r_* = 179.28
Orthorhombic, 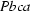

*a* = 9.9881 (11) Å
*b* = 11.8071 (13) Å
*c* = 14.1039 (15) Å
*V* = 1663.3 (3) Å^3^
*Z* = 8
Mo *K*α radiation
μ = 1.01 mm^−1^
*T* = 100 (2) K
0.30 × 0.02 × 0.02 mm

#### Data collection

Bruker SMART APEX diffractometer
Absorption correction: multi-scan (*SADABS*; Bruker, 2007 ▶) *T*_min_ = 0.751, *T*_max_ = 0.980
19044 measured reflections
2430 independent reflections
2123 reflections with *I* > 2σ(*I*)
*R*_int_ = 0.050

#### Refinement

*R*[*F* ^2^ > 2σ(*F* ^2^)] = 0.043
*wR*(*F* ^2^) = 0.089
*S* = 1.22
2430 reflections
113 parameters
All H-atom parameters refined
Δρ_max_ = 0.62 e Å^−3^
Δρ_min_ = −0.28 e Å^−3^

### 

Data collection: *SMART* (Bruker, 2005 ▶); cell refinement: *SAINT* (Bruker, 2005 ▶); data reduction: *SAINT*; program(s) used to solve structure: *SHELXS97* (Sheldrick, 2008 ▶); program(s) used to refine structure: *SHELXL97* (Sheldrick, 2008 ▶); molecular graphics: *ATOMS* (Dowty, 2005 ▶); software used to prepare material for publication: *SHELXL97*.

## Supplementary Material

Crystal structure: contains datablocks I, global. DOI: 10.1107/S1600536808003589/hb2697sup1.cif

Structure factors: contains datablocks I. DOI: 10.1107/S1600536808003589/hb2697Isup2.hkl

Additional supplementary materials:  crystallographic information; 3D view; checkCIF report

## Figures and Tables

**Table 1:** Hydrogen-bond geometry (Å, °)

*D*—H⋯*A*	*D*—H	H⋯*A*	*D*⋯*A*	*D*—H⋯*A*
N1—H1*A*⋯Cl1	0.89 (3)	2.39 (3)	3.2232 (18)	156 (2)
N1—H1*B*⋯Cl1^i^	0.85 (3)	2.34 (3)	3.1862 (18)	173 (2)
N2—H2*A*⋯Cl1	0.86 (3)	2.46 (3)	3.2168 (18)	148 (2)
N2—H2*B*⋯Cl1^ii^	0.86 (3)	2.36 (3)	3.2016 (18)	165 (2)

Symmetry codes: (i) 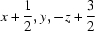; (ii) 
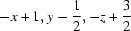
.

**Fig. 1.**
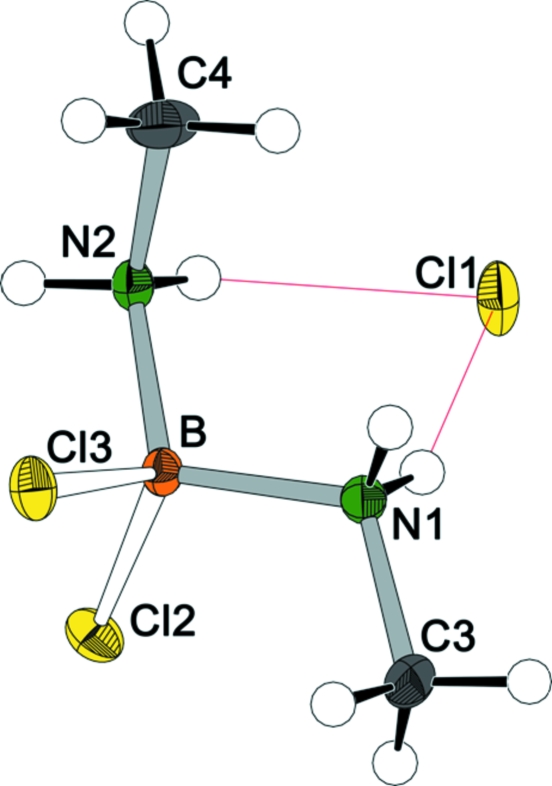
Molecular structure of (I) with displacement ellipsoids drawn at the 50% probability level (arbitrary spheres for the H atoms). Hydrogen bonds are indicated by thin red lines.

**Fig. 2.**
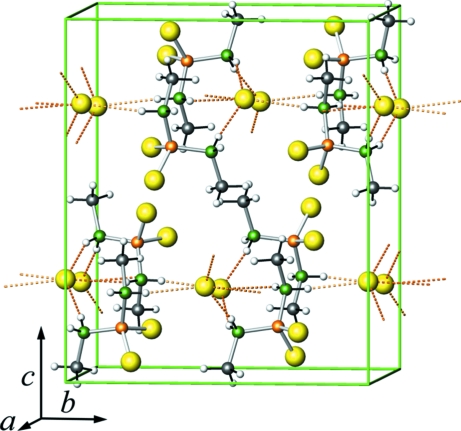
Packing diagram of (I) with hydrogen bonds indicated by dashed lines.

## supplementary crystallographic information

### Comment

Borazonium cations BCl_2_^+^ coordinated by secondary amines *R*_2_NH are very few in number whereas those coordinated by primary amines are more or less unknown. To our knowledge, there has appeared so far no publication dealing with H_3_CNH_2_-coordinated BCl_2_^+^ cations.

We recently published the continuous synthesis of Cl_3_Si-NCH_3_—BCl_2_ (DMTA) by a two-step gas phase synthesis. This reaction proceeds with the formation of solid by-products which are separated from the desired product by filtration. The solid mainly consists of MeNH_3_Cl. Moreover we observed formation of crystalline 2,4,6-trichloro-1,3,5-trimethylborazine, (CH_3_NBCl)_3_ (Weinmann, Nuss *et al.*, 2007). Re-crystallization of the solid from THF/n-pentane now additionally afforded crystals of the title compound, (I) (Fig. 1).

The boron atom in the BCl_2_*L*_2_^+^ cation in (I) is tetrahedrally coordinated by two chlorine atoms Cl2 and Cl3 and two nitrogen atoms N1 and N2 of the methylamine ligands. The smallest angle (N2—B—Cl2) measures 106.38 (13)°, while the biggest (Cl2—B—Cl3) amounts to 113.08 (11)°. The B—Cl bond distances are 1.837 (2) and 1.841 (2) Å whereas the B—N bond lengths measure 1.566 (3) and 1.562 (3) Å. These are values typically found in tetrachloroborate (BCl_4_^-^) or tetraaminoborate (B(N*R*_2_)_4_^-^) anions, respectively.

The chloride counter anions are associated with the cations *via* N—H···Cl hydrogen bonds (Table 1). From Figure 2 it is evident that the anions are each connetcted to four hydrogen atoms, thereby linking three cations. Consequently all the N-bonded H atoms contribute to hydrogen-bond bridges.

A further special feature is the formation of a layered structure in (001) which results from the H-bridge formation. The layers, which are stacked in [001] are connected *via* less polar van-der-Vaals interactions.

### Experimental

[BCl_2_(H_3_CNH_2_)_2_]Cl was obtained as a side-product in amounts < 5% during the continuous synthesis of DMTA. Details of the experimental setup are found elsewhere (Weinmann, Nuss *et al.*, 2007; Weinmann, Kroschel *et al.*, 2007). Re-crystallization of the reaction mixture from THF/n-hexane afforded colourless needles of (I).

### Refinement

All H atoms were found in a difference map and their positions and *U*_iso_ values were freely refined.

### Crystal data

**Table:**

C_2_H_10_BCl_2_N_2_^+^·Cl^–^	*F*_000_ = 736
*M**_r_* = 179.28	*D*_x_ = 1.432 Mg m^−^^3^
Orthorhombic, *P**b**c**a*	Mo *K*α radiation λ = 0.71073 Å
Hall symbol: -P 2ac 2ab	Cell parameters from 5369 reflections
*a* = 9.9881 (11) Å	θ = 2.6–34.8º
*b* = 11.8071 (13) Å	µ = 1.01 mm^−^^1^
*c* = 14.1039 (15) Å	*T* = 100 (2) K
*V* = 1663.3 (3) Å^3^	Needle, colourless
*Z* = 8	0.30 × 0.02 × 0.02 mm

### Data collection

**Table:**

Bruker SMART APEX diffractometer	2430 independent reflections
Radiation source: fine-focus sealed tube	2123 reflections with *I* > 2σ(*I*)
Monochromator: graphite	*R*_int_ = 0.050
*T* = 100(2) K	θ_max_ = 30.0º
ω scans	θ_min_ = 2.9º
Absorption correction: multi-scan(SADABS; Bruker, 2007)	*h* = −14→14
*T*_min_ = 0.751, *T*_max_ = 0.980	*k* = −16→16
19044 measured reflections	*l* = −19→19

### Refinement

**Table:**

Refinement on *F*^2^	Secondary atom site location: difference Fourier map
Least-squares matrix: full	Hydrogen site location: difference Fourier map
*R*[*F*^2^ > 2σ(*F*^2^)] = 0.043	All H-atom parameters refined
*wR*(*F*^2^) = 0.089	*w* = 1/[σ^2^(*F*_o_^2^) + (0.036*P*)^2^ + 0.6817*P*] where *P* = (*F*_o_^2^ + 2*F*_c_^2^)/3
*S* = 1.22	(Δ/σ)_max_ = 0.001
2430 reflections	Δρ_max_ = 0.62 e Å^−^^3^
113 parameters	Δρ_min_ = −0.28 e Å^−^^3^
Primary atom site location: structure-invariant direct methods	Extinction correction: none

### Special details

**Table:**

Geometry. All e.s.d.'s (except the e.s.d. in the dihedral angle between two l.s. planes) are estimated using the full covariance matrix. The cell e.s.d.'s are taken into account individually in the estimation of e.s.d.'s in distances, angles and torsion angles; correlations between e.s.d.'s in cell parameters are only used when they are defined by crystal symmetry. An approximate (isotropic) treatment of cell e.s.d.'s is used for estimating e.s.d.'s involving l.s. planes.
Refinement. Refinement of *F*^2^ against ALL reflections. The weighted *R*-factor *wR* and goodness of fit *S* are based on *F*^2^, conventional *R*-factors *R* are based on *F*, with *F* set to zero for negative *F*^2^. The threshold expression of *F*^2^ > σ(*F*^2^) is used only for calculating *R*-factors(gt) *etc*. and is not relevant to the choice of reflections for refinement. *R*-factors based on *F*^2^ are statistically about twice as large as those based on *F*, and *R*- factors based on ALL data will be even larger.

### Fractional atomic coordinates and isotropic or equivalent isotropic displacement parameters (Å^2^)

**Table:**

	*x*	*y*	*z*	*U*_iso_*/*U*_eq_	
Cl1	0.43797 (5)	0.04596 (4)	0.73581 (4)	0.02091 (13)	
Cl2	0.54279 (5)	−0.21105 (4)	0.55584 (3)	0.01808 (12)	
Cl3	0.82564 (5)	−0.25006 (4)	0.63429 (4)	0.01710 (12)	
B	0.6695 (2)	−0.16781 (17)	0.64305 (15)	0.0120 (4)	
N1	0.70412 (17)	−0.03969 (14)	0.62830 (12)	0.0136 (3)	
H1A	0.630 (3)	−0.002 (2)	0.6429 (17)	0.021 (6)*	
H1B	0.767 (3)	−0.023 (2)	0.6669 (19)	0.024 (7)*	
N2	0.60546 (17)	−0.18659 (14)	0.74288 (12)	0.0137 (3)	
H2A	0.535 (3)	−0.145 (2)	0.7468 (19)	0.030 (7)*	
H2B	0.579 (3)	−0.256 (3)	0.744 (2)	0.028 (7)*	
C3	0.7536 (3)	−0.01034 (19)	0.53109 (16)	0.0212 (4)	
H3A	0.831 (3)	−0.059 (2)	0.519 (2)	0.032 (7)*	
H3B	0.682 (3)	−0.020 (3)	0.488 (2)	0.040 (8)*	
H3C	0.782 (3)	0.066 (2)	0.5310 (19)	0.027 (7)*	
C4	0.6894 (3)	−0.1676 (2)	0.82892 (16)	0.0253 (5)	
H4A	0.636 (3)	−0.178 (2)	0.8830 (19)	0.025 (7)*	
H4B	0.766 (4)	−0.218 (3)	0.831 (2)	0.045 (9)*	
H4C	0.724 (3)	−0.094 (3)	0.825 (2)	0.034 (8)*	

### Atomic displacement parameters (Å^2^)

**Table:**

	*U*^11^	*U*^22^	*U*^33^	*U*^12^	*U*^13^	*U*^23^
Cl1	0.0190 (2)	0.0103 (2)	0.0334 (3)	0.00027 (17)	0.0106 (2)	−0.00070 (18)
Cl2	0.0168 (2)	0.0200 (2)	0.0174 (2)	−0.00113 (18)	−0.00353 (18)	−0.00502 (18)
Cl3	0.0131 (2)	0.0133 (2)	0.0249 (3)	0.00233 (17)	0.00170 (18)	−0.00173 (18)
B	0.0121 (9)	0.0100 (9)	0.0140 (10)	−0.0011 (7)	0.0011 (7)	0.0001 (7)
N1	0.0143 (8)	0.0125 (8)	0.0141 (8)	−0.0001 (6)	0.0007 (6)	−0.0001 (6)
N2	0.0144 (8)	0.0105 (7)	0.0161 (8)	−0.0011 (6)	0.0004 (6)	−0.0003 (6)
C3	0.0308 (12)	0.0155 (10)	0.0173 (10)	−0.0007 (9)	0.0065 (9)	0.0034 (8)
C4	0.0299 (12)	0.0321 (13)	0.0140 (10)	−0.0132 (10)	−0.0023 (9)	0.0022 (9)

### Geometric parameters (Å, °)

**Table:**

B—Cl2	1.837 (2)	N2—H2A	0.86 (3)
B—Cl3	1.841 (2)	N2—H2B	0.86 (3)
B—N2	1.562 (3)	C3—H3A	0.98 (3)
B—N1	1.566 (3)	C3—H3B	0.95 (3)
N1—C3	1.498 (3)	C3—H3C	0.95 (3)
N1—H1A	0.89 (3)	C4—H4A	0.94 (3)
N1—H1B	0.85 (3)	C4—H4B	0.97 (3)
N2—C4	1.492 (3)	C4—H4C	0.93 (3)
			
N2—B—N1	110.33 (15)	C4—N2—H2B	107.4 (18)
N2—B—Cl2	106.38 (13)	B—N2—H2B	106.0 (18)
N1—B—Cl2	109.37 (13)	H2A—N2—H2B	107 (3)
N2—B—Cl3	109.41 (13)	N1—C3—H3A	106.6 (16)
N1—B—Cl3	108.27 (13)	N1—C3—H3B	108.3 (18)
Cl2—B—Cl3	113.08 (11)	H3A—C3—H3B	114 (2)
C3—N1—B	114.71 (16)	N1—C3—H3C	108.7 (16)
C3—N1—H1A	111.9 (16)	H3A—C3—H3C	109 (2)
B—N1—H1A	105.4 (17)	H3B—C3—H3C	110 (2)
C3—N1—H1B	106.6 (18)	N2—C4—H4A	108.8 (17)
B—N1—H1B	107.7 (18)	N2—C4—H4B	112.0 (19)
H1A—N1—H1B	110 (2)	H4A—C4—H4B	110 (3)
C4—N2—B	118.80 (16)	N2—C4—H4C	107.7 (18)
C4—N2—H2A	108.7 (18)	H4A—C4—H4C	112 (2)
B—N2—H2A	108.1 (18)	H4B—C4—H4C	106 (3)

### Hydrogen-bond geometry (Å, °)

**Table:**

*D*—H···*A*	*D*—H	H···*A*	*D*···*A*	*D*—H···*A*
N1—H1A···Cl1	0.89 (3)	2.39 (3)	3.2232 (18)	156 (2)
N1—H1B···Cl1^i^	0.85 (3)	2.34 (3)	3.1862 (18)	173 (2)
N2—H2A···Cl1	0.86 (3)	2.46 (3)	3.2168 (18)	148 (2)
N2—H2B···Cl1^ii^	0.86 (3)	2.36 (3)	3.2016 (18)	165 (2)

Symmetry codes: (i) *x*+1/2, *y*, −*z*+3/2; (ii) −*x*+1, *y*−1/2, −*z*+3/2.
